# Over Ten-Year Survival Rates of Radicular Cyst-Causing Teeth and Factors Affecting Long-Term Outcomes With Apical Microsurgery: A Single-Centre Retrospective Analysis

**DOI:** 10.7759/cureus.89770

**Published:** 2025-08-11

**Authors:** Masaru Ogawa, Satoshi Yokoo, Takahiro Yamaguchi, Keisuke Suzuki, Takaya Makiguchi

**Affiliations:** 1 Oral and Maxillofacial Surgery, and Plastic Surgery, Gunma University Graduate School of Medicine, Maebashi, JPN

**Keywords:** apical microsurgery, long-term outcome, prognostic factors, radicular cyst, tooth survival

## Abstract

Purpose

This study aimed to evaluate the long-term efficacy and identify prognostic factors influencing the survival of teeth with radicular cysts treated by apical microsurgery.

Methods

A total of 212 teeth from 152 patients with histopathologically confirmed radicular cysts who underwent apical microsurgery at Gunma University Hospital between 2009 and 2021 were included. Treatment outcomes were classified as “success” if the treated tooth was preserved and “failure” if the tooth was extracted. Clinical variables showing significant differences in univariate analysis were included in a logistic regression model using forward selection, with treatment outcome as the dependent variable, to identify independent prognostic factors.

Results

The cumulative success rates at two, five, and 12 years were 93.5%, 80.2%, and 76.2%, respectively. Arch type, tooth position, periodontal pocket depth, multiple root canal treatments, apicomarginal bone defects, and perilesional sclerotic signs showed significant differences between the success and failure groups. Multivariate analysis revealed that periodontal pocket depth, apicomarginal bone defects, and tooth position were independent prognostic factors.

Conclusion

The long-term success of apical microsurgery for teeth with radicular cysts is adversely affected by the presence of periodontal pockets ≥ 4 mm, apicomarginal bone defects, and involvement of molars.

## Introduction

Apical microsurgery yields good results for periapical periodontitis and apical granuloma, with a success/survival rate exceeding 85% within a two-year observation period. However, recent studies have shown that the success rate of apical microsurgery for these lesions and survival of the causal tooth decrease over longer observation periods [1]. As short-term follow-up for only one to two years may result in overestimation of the prognosis, studies have documented the factors affecting long-term survival and prognosis [1-4].

Previous studies on apical microsurgery for periapical lesions have included cases of chronic periapical periodontitis, radicular granulomas, and radicular cysts. Radicular cysts, which represent the final pathological condition of chronic periapical periodontitis, are formed when the epithelium encapsulates inflammatory tissue. Therefore, radicular cysts are the most refractory periapical lesions because their pathogenesis differs from that of chronic periapical periodontitis and apical granulomas. No study has specifically examined the effectiveness of apical microsurgery on radicular cyst-causing teeth, that is, the survival rates and prognostic factors.　

This single-centre retrospective study was conducted to determine the long-term survival and prognostic factors for radicular cyst-causing teeth, the most refractory type of apical lesion, treated with apical microsurgery.

## Materials and methods

This study conformed to the Declaration of Helsinki and was approved by the Institutional Research Committee of Gunma University (IRB number: HS2019-144). Written informed consent was obtained from all the enrolled patients using the opt-out method.

Treatment protocol of radicular cyst and apical microsurgery at Gunma University Hospital

**Figure 1:**
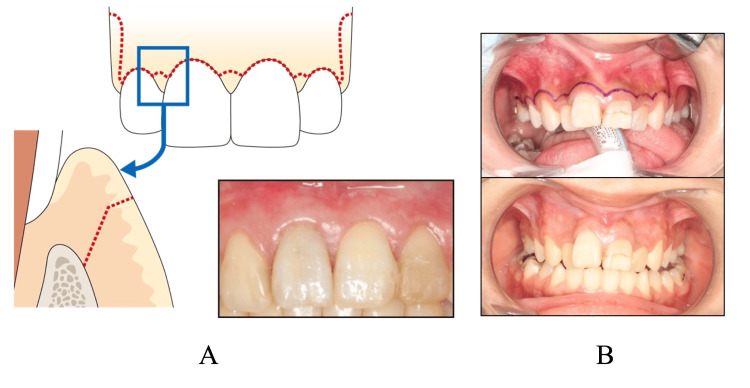
Incision lines A: papilla-based incision and condition three months after surgery; B: luebke-Ochsenbein incision and condition three months after surgery.

**Figure 2:**
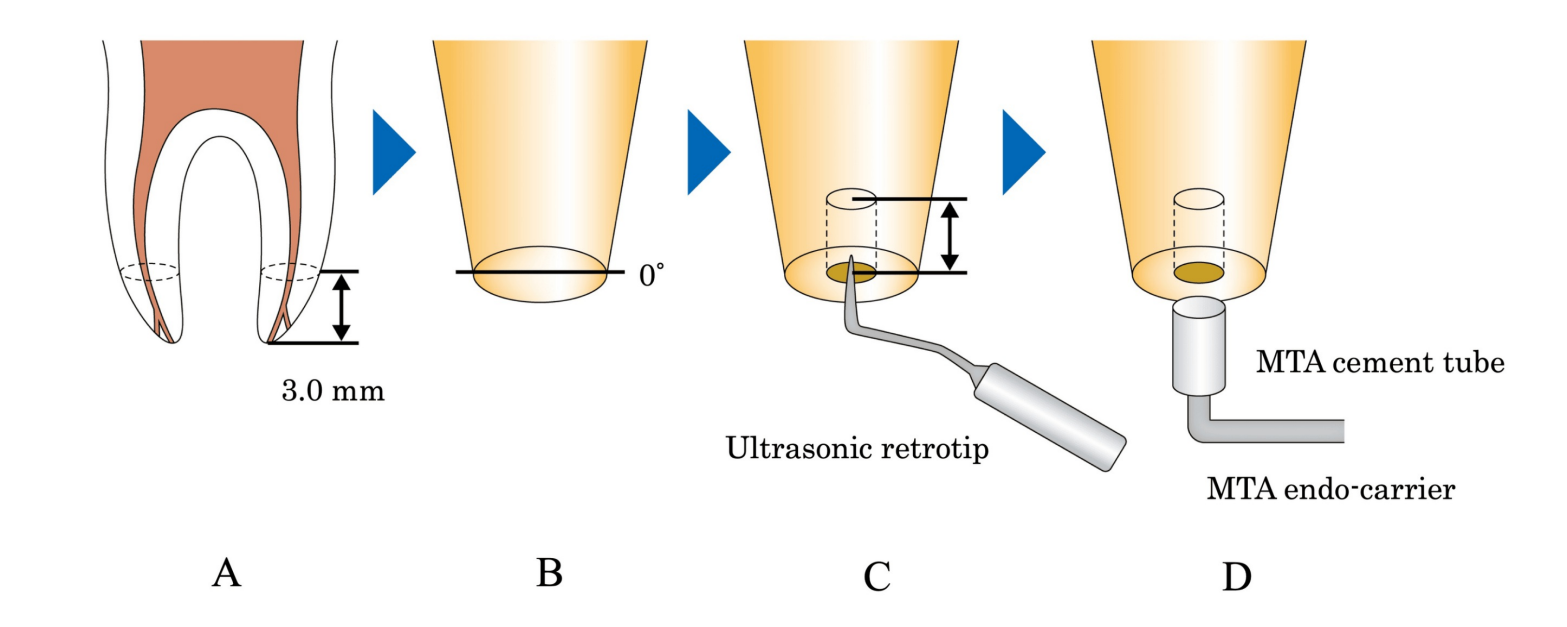
Microscopic apicoectomy and retrograde filling of the root canal with mineral trioxide aggregate (MTA) cement A: apicoectomy range: 3.0 mm; B: bevel angle: 0°, C: retrograde root canal cavity formation with ultrasonic retrotip induction: 3.0 mm; D: retrograde filling of the root canal formation with MTA using an endo-carrier.

**Figure 3:**
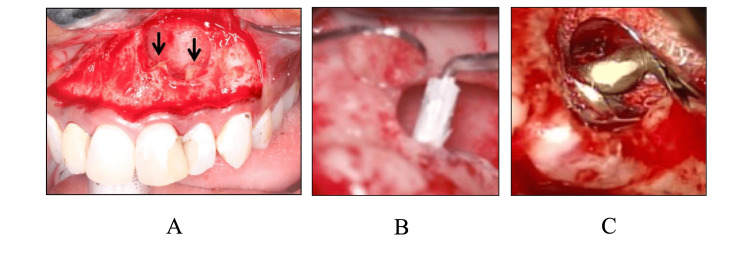
Surgical procedure for the causative teeth A: bone defect after cyst extirpation (arrow: root apex); B: retrograde filling of the root canal with MTA using an endo-carrier after apicoectomy; C: completion of retrograde filling of the root canal.

**Figure 4:**
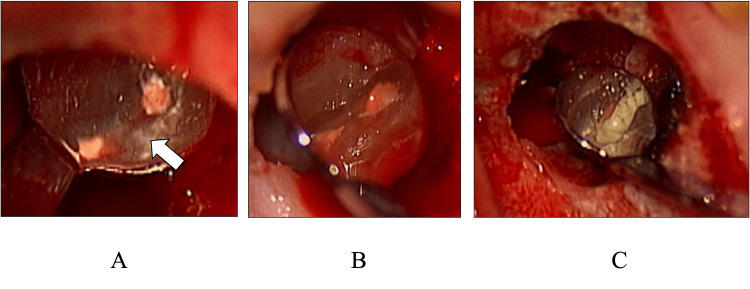
Treatment of the isthmus A: arrow: isthmus; B: preparation of the retrograde root canal cavity, including the isthmus; C: MTA cement filling.

The apical microsurgery method used at Gunma University Hospital, which includes incision, lesion extirpation, retrograde filling of the root canal, and gingival suture, largely adheres to the procedure described by Kim et al. [5] (Table 1 and Figures 1, 2, 3, 4). Surgery is performed under general anaesthesia when the lesion is large, the operative time is predicted to be long, or apical microsurgery involves the molar region. An OME-7000 microscope® (Olympus Corporation©, Japan) used for neurosurgery and vascular anastomosis enables an assistant to work in the same visual field as the operator because of its binocular side-viewing design. Moreover, the microscope is connected to a monitor, making it a valuable tool for educating medical and dental students, house surgeons, and residents. A papilla-based incision [6], a modification of the gingival sulcus incision that conserves the interdental papilla, or the Luebke-Ochsenbein incision is created [7]. An ophthalmological 0° scalpel is particularly useful for gingival incisions because of its size, thinness, flexibility, and blade angle, making it well-suited for creating surgical incisions in a microscopically magnified visual field. To remove the apical ramification and lateral branches, 3 mm of the root apex is resected to achieve a 0° bevel angle of the cut surface, reducing exposure of the dentinal tubules. After apicoectomy, the residual lesion is examined microscopically, and the cut surface fracture, accessory root canal(s), and isthmus are examined microscopically using methylene blue staining. Finally, retrograde root canal filling is performed using mineral trioxide aggregate (MTA) (ProRoot MTA, Dentsply Sirona, New York, USA) cement after ensuring haemostasis and moisture protection. To prevent postoperative gingival trapdoor deformity, concavity, and scarring, the wound is tightly sutured with a thin (6-0) nylon monofilament, as the buried suture used for the skin is inapplicable to gingivoperiosteal flaps. The suture is secured under a microscope. The efficacy of apical microsurgery can be attributed to the following factors: (i) the amount of root apex to be resected can be set; (ii) exposure of the dentinal tubules can be minimised; (iii) a fracture line in the cross-sectional surface of the root apex, untreated root canal, lateral branches, and isthmus can be identified; (iv) the root canal can be tightly sealed with a filling; and (v) lesions around the roots can be removed reliably [5]. Resection of 3 mm of root apex reportedly results in the removal of 98% of the apical ramifications and 93% of the lateral branches [8]. The bevel angle of the cut surface of the exposed dental tubule is 0°. In conventional macroscopic apicoectomy, the bevel angle of the cut surface while preparing the retrograde cavity is 45-60° [9]. In apical microsurgery, the bevel angle of the cut surface of the exposed dentinal tubules is 0°. Under microscopy, a cavity with a bevel angle close to 0° can be prepared using an ultrasonic retrotip. The efficacy of retrotips is attributed to the following: (i) avoidance of perforation of the wall of the root canal by preparing a cavity parallel to the longitudinal axis of the tooth and (ii) preparation of a cavity with a depth ≥ 3 mm, which is needed to avoid dislodgement of the retrograde root canal-filling agent. We use biocompatible MTA cement [10], a retrograde root canal-filling agent with good cavity-sealing properties attributed to its main ingredients, which include inorganic oxides such as CaCO3 and SiO2. Moreover, it has a favourable sealing ability, even when exposed to blood, and can reportedly induce the differentiation of cementoblasts [11]. Therefore, currently, MTA cement may be the ideal retrograde root canal filling material after apicoectomy [12].

**Table 1:** Summary of apical microsurgery

	Conventional	Kim et al.'s method	Our institution’s method
Length of root-end resection	8-10 mm	3-4 mm	3 mm
Bevel angle degree	45-65°	0-10°	0°
Inspection of resected root surface	None	In general	In general
Isthmus identification and treatment	Impossible	In general	In general
Root-end preparation	Seldom inside the canal	Always within the canal	Always within the canal
Root-end resection instrument	Bur	Ultrasonic tips	Ultrasonic tips
Root-end filling material	Amalgam	MTA cement	MTA cement
Suture	4-0 silk	5-0, 6-0 monofilament	6-0 monofilament
Suture removal after surgery	7 days	2-3 days	7 days

Analysis of comprehensive clinical outcomes in apical microsurgery

This study enrolled 212 teeth from 152 patients with histopathologically diagnosed radicular cysts treated with the Partch II method and apical microsurgery at the Department of Oral and Maxillofacial Surgery, Gunma University Hospital, between 2009 and 2021. The clinical outcome was designated as a "success" if the tooth indicated for apical microsurgery was preserved throughout the follow-up period, regardless of radiographic or clinical signs of healing, and as a "failure" if the tooth was extracted. In cases where the lesion involved multiple teeth and apical microsurgery was performed on more than one tooth, each treated tooth was considered as an individual unit of analysis. The endpoints were set to the date of causal tooth extraction or the date of the last observation. Cases in which the tooth remained functional at the last clinical follow-up were treated as censored observations. The two, five, and 12-year cumulative survival rates (disease-specific tooth survival) of teeth that underwent apical microsurgery were calculated using the Kaplan-Meier method.

Analysis of prognostic factors in apical microsurgery

**Figure 5:**
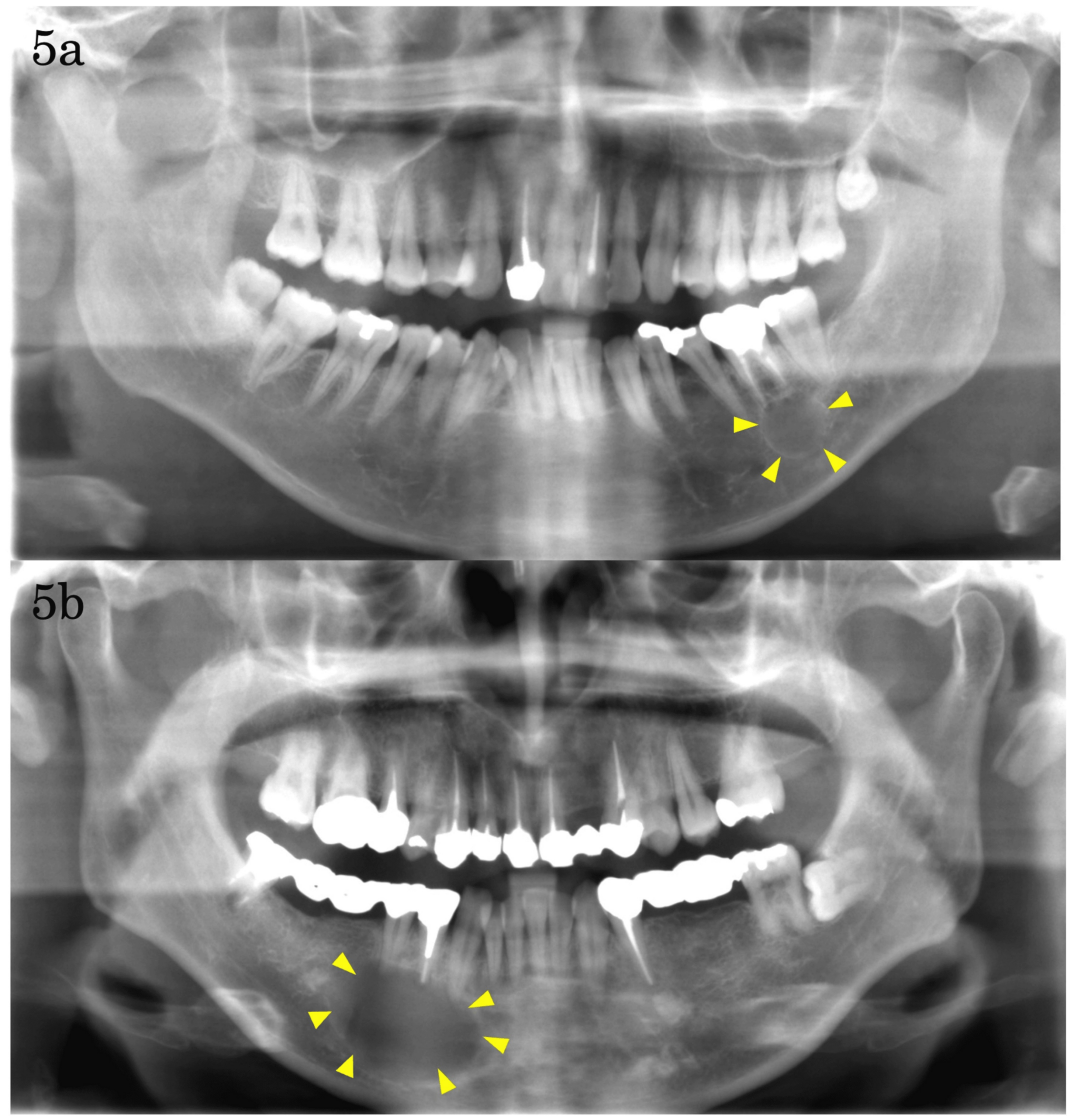
Assessment of lesion size using panoramic radiographs 5a: Cyst involving one or two teeth; 5b: Cysts involving three or more teeth.

**Figure 6:**
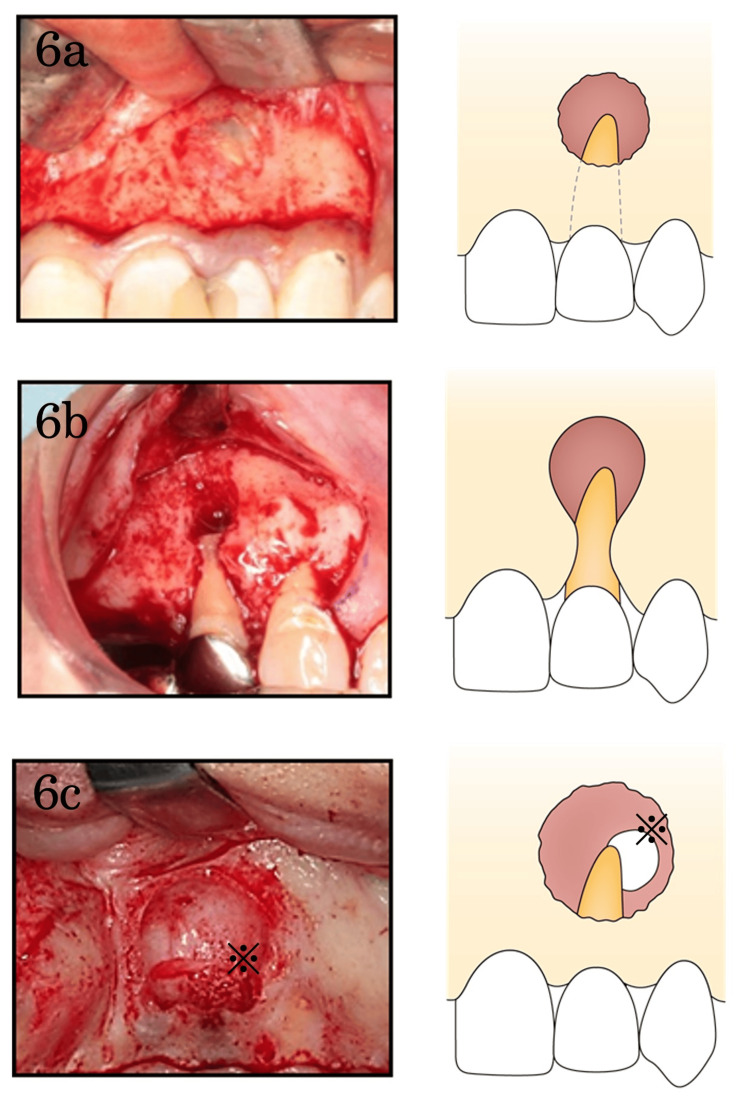
Classification of cortical bone defects 6a: Cortical bone fenestration: one-sided defect of the buccal, lingual, or palatal cortical bones with the cervical bone of the tooth remaining intact; 6b: Apicomarginal bone defect: continuous bone defect from the lesion in the apical region to the tooth cervix; 6c: Through-and-through bone defect: involvement of both buccal and lingual/palatal cortical bones (*palatal bone defect).

**Figure 7:**
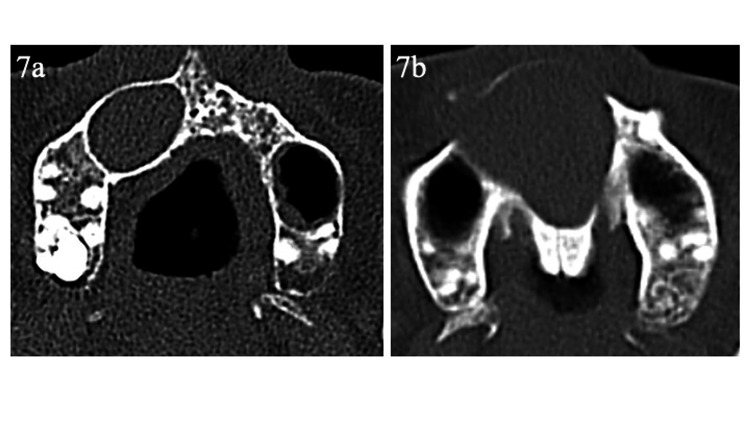
Representative computed tomography (CT) images of perilesional sclerotic signs 7a: Radicular cyst with a well-defined radiopaque sclerotic rim around the lesion, indicating a positive perilesional sclerotic sign; 7b: Radicular cyst without surrounding radiopaque changes, indicating a negative perilesional sclerotic sign.

The relationship between the clinical outcomes and clinical factors was statistically analysed in 212 teeth to identify the prognostic factors for apical microsurgery for radicular cyst-causing teeth. The clinical factors were categorised into patient, dental, pathological, operative, and osseous variables. The patient variables included age, sex, history of diabetes mellitus, and administration of anticoagulant or steroid therapy. The dental variables were arch type (maxillary/mandibular), tooth position (anterior/posterior), post in the dental root (presence/absence), periodontal pocket depth (< 4 mm/≥ 4 mm), history of multiple root canal treatment, and the preoperative root canal filling condition (adequate/inadequate).In this study, “anterior” referred to incisors and canines, and “posterior” included premolars and molars. Assessment of periodontal pocket depth was based on the Clinical Guidelines for Periodontal Treatment 2022 of The Japanese Society of Periodontology, which defines moderate to severe periodontitis as a pocket depth of ≥ 4 mm [13]. The preoperative condition of the root canal filling was assessed according to the study of Sjögren et al. [14], who reported satisfactory outcomes when the canal was filled within 2 mm of the root apex. Thus, cases in which the root canal filling agent was 0-2 mm from the root apex were considered adequate, and those with under- or overfilling of ≥ 2 mm were considered inadequate. The pathological variables included the presence of preoperative pain, fistula, and lesion size. Lesion size was classified based on the number of teeth involved, distinguishing between cysts involving three or more teeth and those with one or two teeth (Figure 5). The operative variables included the type of anaesthesia and the number of teeth treated simultaneously (single/multiple). The osseous variables were cortical bone fenestration, apicomarginal bone defects, through-and-through bone defects, and perilesional sclerosis signs, which were defined as follows (Figure 6): (i) cortical bone fenestration, in which only one side of the buccal, lingual, or palatal cortical bone is defective, but the cervical bone of the tooth is intact; (ii) apicomarginal bone defect, in which the bone is defective continuously from the apical lesion to the tooth cervix; and (iii) through-and-through bone defect in which both the buccal and lingual/palatal cortical bones are defective. Sclerotic changes in the bone around the cyst on computed tomography (CT) were designated as perilesional sclerotic signs (Figure 7). CT image assessments were performed by two oral surgeons and one radiology specialist.

Using the above-mentioned binary values as explanatory variables and success or failure as the objective variable, comparisons between the success and failure groups were performed using the χ2 test for independence. Next, clinical factors with significant differences in the χ2 test were used as explanatory variables and success or failure as the objective variable, and logistic regression analysis with a forward-selection method was conducted to identify the prognostic factors in apical microsurgery. The extracted explanatory variables exhibited variance inflation factors ≤ 10, confirming the absence of multicollinearity. Statistical analyses were performed using SPSS for Windows version 25 (IBM Corp., Armonk, NY, USA). P-values < 0.05 were considered statistically significant.

## Results

Patient background

Table 2 shows the background of patients with radicular cysts who underwent apical microsurgery. The median patient age was 47 years (12-81 years). Eighty-two patients were men, and 130 were women. The most common sites of involvement were the maxillary anterior teeth (n = 102), followed by the mandibular molars (n = 45), maxillary molars (n = 41), and mandibular anterior teeth (n = 24), respectively. At the end of the observation period, 198 (93.4%) teeth were preserved, and 14 (6.6%) teeth were extracted. The reasons for extraction were root fractures in seven teeth and recurrence of the radicular cyst in seven teeth. The postoperative observation period ranged from two to 160 months (median: 24 months).

**Table 2:** Patients' background

Factors	Number of teeth (n = 212)
Sex	
Men	82
Women	130
Location	
Maxillary anterior	102
Maxillary molar	41
Mandibular anterior	24
Mandibular molar	45
Outcome	
Teeth preserved (Success)	198
Teeth extracted (Failure)	14
Root fracture	7
Recurrence of lesion	7
Age (years), Median (range)	47 (12-81)
Follow-up (months), Median (range)	24 (2-160)

Prognostic factors in apical microsurgery

The results of the comparison of the clinical factors between the success and failure groups are shown in Table 3. According to the univariate analysis, arch type (χ² = 5.931, p = 0.015, df = 1, Cramér’s V = 0.167), tooth position (χ² = 5.922, P = 0.015, df = 1, Cramér’s V = 0.167), periodontal pocket depth (χ² = 42.337, P < 0.001, df = 1, Cramér’s V = 0.447), multiple root canal treatments (χ² = 11.959, P = 0.003, df = 1, Cramér’s V = 0.238), apicomarginal bone defects (χ² = 30.450, P < 0.001, df = 1, Cramér’s V = 0.420), and perilesional sclerotic signs (χ² = 8.456, P = 0.003, df = 1, Cramér’s V = 0.200) differed significantly between the success and failure groups. Multivariate analysis using these six parameters that were statistically significant in the univariate analysis showed that periodontal pocket depth (P < 0.001), apicomarginal bone defect (P < 0.001), and tooth position (P = 0.034) were independent prognostic factors for apical microsurgery.

**Table 3:** Univariate and multivariate analyses of factors related to the treatment outcome

					Univariate analysis	Multivariate analysis		
	Explanatory variables (n = 212)		Success (n = 198)	Failure (n = 14)	P value	P value	Odds ratio	95% CI
Patients’ variables	Age	< 47	94	8	0.484			
		≥ 47	104	6				
	Sex	Man	78	4	0.422			
		Woman	120	10				
	Diabetes mellitus	No	190	14	0.573			
		Yes	8	0				
	Anticoagulant therapy	Not administered	194	14	0.759			
		Administered	4	0				
	Steroid therapy	Not administered	197	14	0.934			
		Administered	1	0				
Dental variables	Arch type	Maxilla	138	5	0.015			
		Mandible	60	9				
	Tooth position	Anterior	122	4	0.015	0.034	5.065	1.135-22.610
		Posterior	76	10				
	Post	Absence	114	6	0.283			
		Present	84	8				
	Periodontal pocket depth	< 4 mm	179	4	< 0.001	< 0.001	10.516	2.596-42.597
		≥ 4 mm	19	10				
	Multiple root canal treatment	No	191	10	0.003			
		Yes	7	4				
	Condition of root canal filling	Adequate	184	13	0.654			
		Inadequate	14	1				
Pathological variables	Pain	Absence	152	8	0.113			
		Present	46	6				
	Fistula	Absence	183	13	0.715			
		Present	15	1				
	Lesion size	< 3 teeth	107	8	0.822			
		≥ 3 teeth	91	6				
Operative variables	Anaesthesia	General anaesthesia	143	3	0.762			
		Local anaesthesia	55	11				
	Number of treated teeth	Single	80	9	0.080			
		Multi	118	5				
Osseous variables	Cortical bone defect	Absence	63	4	0.531			
		Present	135	10				
	Apicomarginal bone defect	Absence	188	6	<0.001	<0.001	18.303	4.034-83.046
		Present	10	8				
	Through-and-through bone defect	Absence	171	12	0.598			
		Present	27	2				
	Perilesional sclerotic signs	Absence	191	10	0.003			
		Present	7	4				

Comprehensive clinical outcomes of apical microsurgery

**Figure 8:**
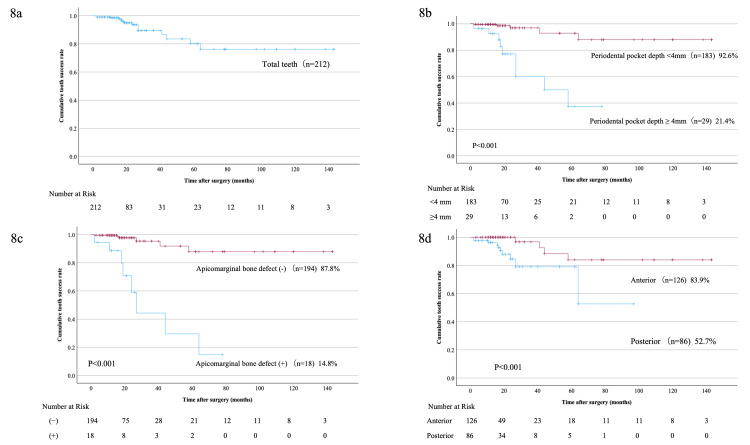
Cumulative survival rate 8a: The 2-, 5-, and 12-year cumulative success rates of the 212 teeth were 93.5%, 80.2%, and 76.2%, respectively; 8b: The 70-month survival rate of teeth with a periodontal pocket depth < 4 mm was 92.6% and that for teeth with a probing depth ≥ 4 mm was 21.4% (P < 0.001); 8c: The 70-month tooth survival was 87.8% in the negative and 14.8% in the positive apicomarginal bone defect groups (P < 0.001); 8d: The 90-month tooth survival rate was 83.9% for anterior teeth and 52.7% for posteriors (P < 0.001).

Figure 4 shows the cumulative success rates of apical microsurgery. The two, five, and 12-year cumulative success rates of the 212 teeth were 93.5%, 80.2%, and 76.2%, respectively (Figure 8a). The 70-month survival rates of teeth with a periodontal pocket depth < 4 mm and ≥ 4 mm were 92.6% and 21.4%, respectively (P = 0.001) (Figure 8b). The 70-month tooth survival was 87.8% in the negative and 14.8% in the positive apicomarginal bone defect groups (P < 0.001) (Figure 8c). Moreover, the 90-month tooth survival rate was 83.9% for anterior teeth and 52.7% for molars (P < 0.001) (Figure 8d).

## Discussion

In this study, we made two important clinical observations. First, apical microsurgery performed for radicular cysts involving 212 teeth yielded a two-year survival rate of 93.5%, five-year survival rate of 80.2%, and 12-year survival rate of 76.2%, comparable to previously reported survival rates for teeth with periapical periodontitis. Second, the prognostic factors affecting the long-term results of apical microsurgery for radicular cyst-causing teeth were marginal periodontitis with periodontal pocket depth ≥ 4 mm, apicomarginal bone defect, and molar involvement. Radicular cyst size was not a prognostic factor for causative tooth survival.

A recent study based on a two-year observation period reported a tooth survival rate of > 90% after apical microsurgery [3], whereas Truschnegg et al. [1] reported a survival rate of 79% after long-term observation of 13 years. Long-term studies are essential for accurate prognostication, as short-term observations of one to two years may overestimate the outcomes. All previous studies have examined all apical lesions, not just the radicular cyst, the ultimate stage of chronic periapical periodontitis, and the most refractory lesion. The present study showed that apical microsurgery is an effective technique to prolong the survival of radicular cyst-causing teeth. The variability in methods for assessing the efficacy of apical microsurgery complicates comparisons across studies. Post-apicoectomy evaluations generally use a combination of clinical and radiographic assessments to determine success rates, as shown by Molven et al. [15]. However, in large radicular cysts, scar formation will inevitably accompany osseous healing of the extraction cavity. Since complete healing is rare for large radicular cysts, this criterion is unsuitable for assessing the condition of teeth and bone treated with apical microsurgery. Therefore, it is reasonable to consider the survival (residual) rate, not the success (healing) rate, of the causative tooth as the endpoint of this study, as it represents the most important aim of radicular cyst management.

This study identified periodontal pocket depth ≥ 4 mm as a prognostic factor. In cases where periodontal pocket depth ≥ 4 mm was present in the radicular cyst-causing tooth, the 24-month survival rate after apical microsurgery was 78.2%, but the 70-month survival rate was significantly lower (21.4%). The presence of deep periodontal pockets indicates a source of bacterial infection. This nidus of infection may not only compromise periapical healing but can also lead to a significant loss of periodontal attachment in the long term, as observed in teeth that do not heal after apical microsurgery. Lui et al. [16] reported that teeth with a probing depth ≤ 3 mm exhibited successful healing. Kim et al. [17] noted that the success rate of combined endodontic-periodontal lesions was 77.5% over a two to five-year follow-up period compared to 95.2% for apical lesions. Song et al. [18] found that the five-year cumulative survival rate was 87.3% for patients with only an apical lesion, which decreased to 77.5% for combined endodontic-periodontal lesions. Before surgery, patients must be informed that long-term survival beyond five years is unlikely in over half of cases with concomitant periodontal disease and radicular cyst, even with apical microsurgery. A comprehensive assessment of the periodontal disease must be performed before surgical intervention, using both preoperative probing depth measurements and cone-beam CT-assisted alveolar bone evaluation.

This study extracted eight of 18 teeth with apicomarginal bone defects, and five had postoperative root fractures. Merino [19] reported that an apicomarginal bone defect reduced the success rate of endodontic surgery by 37%, making it the most critical prognostic factor for poor outcomes. Grimoud et al. [20] found a high rate of alveolar bone dehiscence in this region, suggesting that an apicomarginal bone defect can be considered a complication of alveolar bone dehiscence and an apical lesion-induced buccal bone defect. Alveolar bone dehiscence may result from anatomical and morphological complications, thin cortical bone, and secondary functional factors such as occlusal load. Vertical root fractures with alveolar bone dehiscence are caused by decreased resistance to occlusal pressure due to a partial periodontal ligament defect [21,22]. Apical microsurgery in teeth with alveolar bone dehiscence creates a surgical bone defect in addition to comorbid bone defects, further reducing the ability to resist occlusal pressure and increasing the risk of root fracture. Radicular cysts, involving the most bone removal among apical lesions, increase the risk of tooth fracture. Therefore, close attention should be paid to managing postoperative occlusion in patients with apicomarginal bone defects. Goyal et al. [23] reported a 90% healing rate for apical microsurgery with guided tissue regeneration (GTR) in cases with apicomarginal bone defects, highlighting the critical role of GTR in treatment. GTR application is anticipated to prevent epithelial and fibroblast invasion in the periodontal pocket, inhibit scar formation, and facilitate osseous healing after radicular cyst extirpation.

This study found that the survival rate after apical microsurgery was lower in molars than in anterior teeth, similar to von Arx et al.’s study [24]. This may be attributed to the thicker cortical bone, comparably difficult surgical access in the molars, and reduced visibility compared to the anterior teeth. The more complex molar root canal anatomy may contribute to a lower success rate. A greater prevalence of the isthmus and lateral canals has been documented in the molars than in the anterior teeth [25]. However, different results have been reported regarding the effect of tooth position on success rates. In a meta-analysis by Tsesis et al. [26], tooth position influenced the success rate only in cases where the traditional technique was used, whereas no effect was noted with apical microsurgery. This meta-analysis revealed fewer cases of apical microsurgery in molars than in anterior teeth. It is also possible that the difference in techniques between surgeons from different centres may have had an effect. The present study can be considered more accurate than other research because its results were obtained from procedures conducted by a single surgeon using the same technique at a single centre.

For clinical application, this study categorised radicular cyst size based on the number of teeth within the cyst, finding no correlation between cyst size and tooth survival after apical microsurgery. von Arx et al. [27] also demonstrated no difference in success rates between lesion size ≤ 5 and > 5 mm. Similarly, Kim et al. [28] reported that the lesion size did not affect the success rate of apical microsurgery. Conversely, other studies have reported that the smaller the lesion size, the more successful the healing after apical microsurgery [29,30]. This is because smaller lesions are extirpated more easily, and the risk of residual lesions is lower. The present study was limited to the most difficult-to-treat radicular cysts and found that cyst size did not affect the poor prognosis of radicular cyst-causing teeth, even though 97 of 212 teeth (46%) had large cysts involving three or more teeth. Using a microscope for periapical curettage enhances reliability, potentially preventing residual lesions and recurrence, which explains the favourable outcomes, irrespective of cyst size.

This study has several limitations. First, the definition of treatment success was based solely on tooth retention. While this approach ensures objectivity and reproducibility of evaluation, it may not fully reflect the clinical resolution of the lesion, which was not separately assessed in this study. In addition, given the long follow-up period, the potential influence of follow-up bias must also be considered when interpreting the results. Second, the patients' oral hygiene was not assessed. Periodontal disease is a prognostic factor (periodontal pocket depth ≥ 4 mm and apicomarginal bone defect) for apical microsurgery and is associated with the individual’s oral hygiene status. It would be beneficial if future studies included parameters reflecting the oral hygiene status. Third, this study was a retrospective analysis based on the patients' medical records. The survival rates of teeth undergoing conventional apical surgery and endodontic treatment were obtained from other studies. To validate this surgical method, it is necessary to compare our survival rates with conventional apical surgery and endodontic treatment and conduct prospective clinical trials.

## Conclusions

Apical microsurgery was performed for radicular cyst-causing teeth, demonstrating favorable long-term outcomes. The prognostic factors affecting the long-term results of apical microsurgery were the presence of periodontal disease, apicomarginal bone defect, and molar involvement. In this study, prognostic factors were analyzed only for the teeth that caused radicular cysts, which are the final form and most refractory chronic apical periodontitis. Interestingly, the prognostic factors were the same as those in previous reports that analysed all chronic periapical lesions. This is the first study to report on the efficacy of apical microsurgery, especially in radicular cyst-causing teeth.
